# A Metabolism-Guided Framework for Selecting Structural Modification or Nano-Delivery Strategies for Oral Rutin

**DOI:** 10.3390/ijms27156543

**Published:** 2026-07-23

**Authors:** Ting He, Ying Wang, Fei Yan, Bei-Sheng Fan, Rui-Jia Fu, Ding-Qiao Xu, Jie Yang, Yu-Ping Tang

**Affiliations:** Key Laboratory of Shaanxi Administration of Traditional Chinese Medicine for TCM Compatibility, Shaanxi University of Chinese Medicine, Xianyang 712046, China

**Keywords:** rutin, gut microbiota, prodrug, structural modification, nanodelivery system, bioavailability, metabolism-guided framework

## Abstract

Rutin is a flavonoid glycoside with well-documented antioxidant and anti-inflammatory activities in preclinical models, but its oral bioavailability is poor due to extensive presystemic metabolism. Following oral administration, intact rutin reaches the colon largely unabsorbed, where gut microbiota convert it to quercetin aglycone, which is further metabolized to circulating conjugated metabolites and local phenolic acid derivatives. Accumulating evidence, derived predominantly from preclinical studies, suggests this metabolic activation can be usefully conceptualized as a microbiota-activated prodrug system. This challenges conventional formulation strategies that only increase parent compound exposure. In this review, we propose a metabolism-oriented conceptual framework for oral rutin delivery design. We compare nano-delivery systems and structural modifications strategies across pharmacokinetics, metabolic regulation, safety, manufacturability, and translational feasibility. Nano-delivery systems are functionally reclassified into four absorption, distribution, metabolism, excretion (ADME) -oriented categories based on their primary pharmacokinetic objectives. While numerous preclinical studies report improved bioavailability and efficacy, human metabolite-resolved pharmacokinetic data remain extremely limited, and no European Food Safety Authority (EFSA) -authorized health claims exist for rutin. This review provides a new conceptual framework for selecting oral rutin delivery strategies based on metabolic objectives rather than solely on improving solubility or systemic exposure.

## 1. Introduction

Rutin (Quercetin-3-*O*-rutinoside) is a naturally occurring flavonoid glycoside widely distributed in Sophora japonica, buckwheat, citrus fruits, tea, and various medicinal plants. Owing to its antioxidant, anti-inflammatory, vasoprotective, neuroprotective, and metabolic regulatory activities, rutin has attracted extensive attention in functional foods, nutraceuticals, and pharmaceutical research [1]. Experimental studies have suggested potential therapeutic applications in cardiovascular diseases, diabetes, neurodegenerative disorders, inflammatory bowel disease, and cancer [2]. However, despite its broad pharmacological potential, the clinical translation of rutin remains severely limited. Conventional studies almost universally attribute this limitation to its poor oral bioavailability, which is associated with low aqueous solubility, poor intestinal permeability, and extensive first-pass metabolism. Consequently, numerous delivery strategies, including nano-delivery systems, phospholipid complexes, cyclodextrin inclusion systems, and structural modification approaches, have been developed to improve its absorption and systemic exposure [3,4]. Accumulating evidence, derived predominantly from preclinical studies, suggests that the oral fate of rutin is far more complex than a simple solubility problem. Due to the presence of the rutinose moiety, intact rutin is poorly absorbed in the small intestine and largely reaches the colon, where it undergoes gut microbiota-mediated deglycosylation to quercetin. Quercetin is subsequently subjected to extensive phase II metabolism, generating glucuronidated, sulfated, and methylated conjugates that constitute the dominant circulating species in vivo [5]. Therefore, many systemic biological effects traditionally attributed to rutin may be mediated by quercetin conjugates and downstream phenolic metabolites rather than the parent compound itself. Notably, intact rutin may still exert direct pharmacological effects at local gastrointestinal sites. This metabolism-dependent activation process offers a useful reframing of rutin pharmacology. In this context, rutin can be usefully conceptualized as a microbiota-activated prodrug system, rather than merely a poorly soluble flavonoid, which challenges conventional solubility-focused formulation strategies. Importantly, different diseases may require distinct active molecular forms. Intestinal disorders may benefit primarily from local exposure to intact rutin and microbial metabolites, whereas systemic metabolic and cardiovascular diseases are more likely associated with circulating quercetin conjugates. Accordingly, the major challenge in oral rutin delivery is not only to increase apparent bioavailability, but also to regulate the generation, distribution, and exposure of active species. These findings also challenge current formulation evaluation criteria. Most existing studies assess delivery systems primarily based on total plasma exposure, while insufficient attention has been paid to metabolite composition, tissue distribution, and translational relevance. Moreover, although extensive preclinical evidence has been reported, rutin currently lacks authorized health claims from the EFSA, highlighting the gap between experimental findings and clinical or regulatory validation. Human metabolite-resolved ADME data also remain extremely limited. Although numerous reviews have summarized the pharmacological activities and formulation strategies of rutin, most remain descriptive and continue to focus predominantly on solubility enhancement. Few reviews have systematically discussed the dominant role of quercetin conjugates in systemic circulation or established a metabolism-guided framework for selecting between nano-delivery and structural modification strategies. Therefore, this review systematically discusses the ADME characteristics and metabolic fate of rutin after oral administration, with a focus on the prodrug perspective. Particular emphasis is placed on gut microbiota-mediated biotransformation, phase II conjugation, enterohepatic circulation, and clinical translation. Furthermore, nano-delivery and structural modification strategies are comparatively evaluated based on pharmacokinetic improvement, active species regulation, safety, manufacturability, and translational feasibility, with the aim of proposing a metabolism-guided conceptual framework for rational oral rutin formulation design.

## 2. Pharmacological Action

Rutin is a natural flavonoid glycoside that exhibits multiple pharmacological effects, primarily centered on antioxidant, anti-inflammatory, metabolic regulation, and vascular protection properties [6,7,8]. It shows therapeutic potential against cancers, cardiovascular and cerebrovascular diseases, metabolic disorders and neurodegenerative diseases by targeting their underlying pathological mechanisms.

In terms of antioxidant activity, rutin scavenges free radicals, chelates metal ions, and inhibits lipid peroxidation, thereby alleviating oxidative stress [9]. Its anti-inflammatory action involves suppression of nuclear factor-κB (NF-κB) and mitogen-activated protein kinase (MAPK) signaling pathways, reducing the release of pro-inflammatory cytokines such as tumor necrosis factor-α and interleukin-6 [10]. In the cardiovascular system, rutin improves endothelial function, inhibits platelet aggregation, and reduces vascular permeability, showing protective effects against hypertension, atherosclerosis, and myocardial ischemia–reperfusion injury [11,12,13]. In metabolic disorders, rutin enhances insulin sensitivity, inhibits α-glucosidase activity, and ameliorates dyslipidemia, indicating potential benefits for diabetes and its complications [14]. In the nervous system, rutin and its metabolites inhibit amyloid-β aggregation, protect dopaminergic neurons, and attenuate neuroinflammation, exhibiting neuroprotective effects in models of Alzheimer’s and Parkinson’s diseases [15,16].

However, the above analysis raises a critical question: if intact rutin itself is poorly absorbed into the systemic circulation, why are significant systemic pharmacological effects consistently observed in numerous animal models. This apparent contradiction between low parent-compound bioavailability and measurable systemic bioactivity prompts has prompted investigation into the relative contributions of different molecular forms. Accumulating evidence, derived predominantly from preclinical studies, indicates that systemic effects of orally administered rutin are mediated largely by its metabolites. Notably, intact rutin may still exert direct local effects in the gastrointestinal tract, where high local concentrations are achieved after oral dosing. Its metabolites are increasingly recognized as the key entities responsible for the in vivo pharmacological effects [17].

## 3. Metabolic Transformation Pathways of Rutin

### 3.1. Microbial Deglycosylation: Conversion to Quercetin Aglycone

The rutinose disaccharide moiety is the primary determinant of rutin’s poor oral absorption. Due to its high polarity (log*P* = −1.97), large molecular weight (610.5 Da), and extensive hydrogen bonding capacity, intact rutin cannot permeate the intestinal epithelium via passive diffusion [18,19]. Consequently, its absorption is largely dependent on hydrolysis by the gut microbiota. Studies have shown that rutin, which possesses a disaccharide structure, undergoes sequential removal of rhamnose and glucose under the action of intestinal microbiota, yielding quercetin. Specifically, α-L-rhamnosidase secreted by gut microbes first acts on rutin to remove the terminal rhamnose, generating isoquercitrin. This intermediate is subsequently hydrolyzed by β-D-glucosidase, releasing the aglycone quercetin [20]. Notably, α-L-rhamnosidase activity represents the rate-limiting step in rutin metabolic activation, and its abundance varies considerably across individuals due to differences in gut microbiota composition [21], which may contribute substantially to interindividual variability in rutin response.

### 3.2. Phase II Conjugation: Further Modification of Quercetin

**Figure 1:**
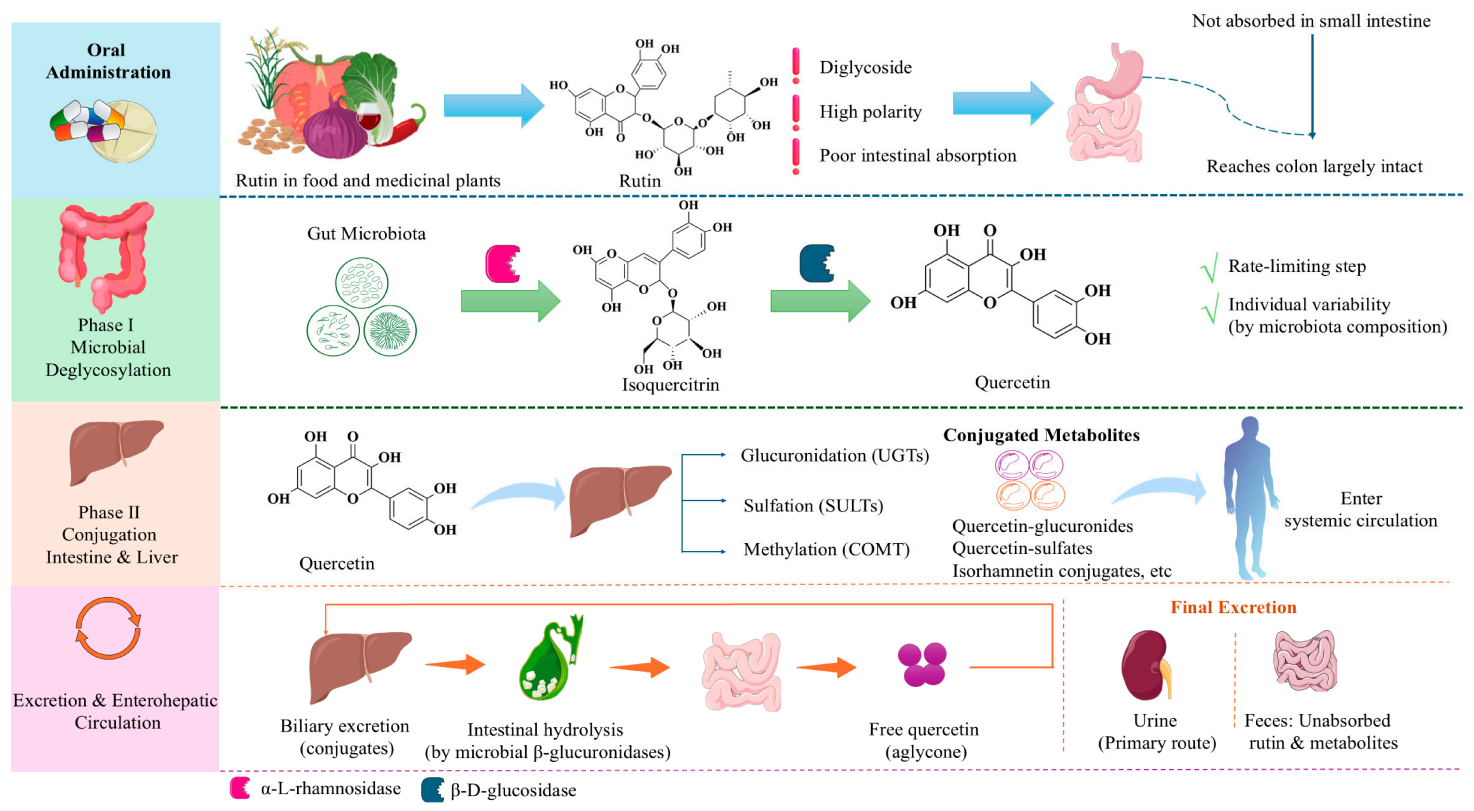
Schematic diagram of the complete in vivo transformation pathway and mechanism of rutin. Solid thick blue arrows: General flow of substance transport and metabolic progression. Solid green arrows: Stepwise phase I deglycosylation reactions catalyzed by gut microbiota. Light blue curved arrows: Portal absorption and hepatic delivery of aglycone. Solid orange arrows: Excretion, reabsorption and enterohepatic circulation processes.

The released quercetin is rapidly absorbed in the small intestine and colon and immediately undergoes extensive phase II metabolism in enterocytes and hepatocytes, primarily via three pathways: glucuronidation, sulfation, and methylation. These conjugation reactions markedly increase the water solubility of the metabolites, facilitating their excretion via urine and bile. Based on metabolite profiling in rats, quercetin glucuronides, sulfates, and methylated products represent the predominant circulating species. Notably, some metabolites may undergo enterohepatic circulation, thereby prolonging their retention time in the body [22]. Figure 1 shows a schematic diagram of the proposed in vivo metabolic pathway of rutin.

### 3.3. Enterohepatic Circulation and Excretion Characteristics

A fraction of conjugated metabolites can be excreted into the bile and re-enter the intestinal lumen. In the intestine, microbial β-glucuronidases hydrolyze these conjugates to regenerate free quercetin. The released aglycone can then be reabsorbed into the systemic circulation, forming an enterohepatic recycling process [23]. This recycling may contribute to the prolonged residence time of rutin-derived metabolites in vivo, which could help explain why once-daily dosing is often sufficient for therapeutic effects. Unabsorbed parent rutin and its metabolites are primarily excreted via feces. Only a small proportion of the administered dose, reported to be approximately less than 10% in some studies, is recovered in urine in the form of conjugated metabolites [24]. This excretion pattern suggests that, following oral administration, exposure to rutin and its metabolites is largely confined to the gastrointestinal tract and liver, with relatively limited systemic availability.

### 3.4. Differences in the Demand for Metabolites Among Various Diseases

Rutin, quercetin, and their metabolites exhibit distinct pharmacokinetic profiles, tissue distribution patterns, and target specificities, which may determine their suitability for different therapeutic applications. In vivo data from rat studies demonstrate that intact rutin barely crosses the blood–brain barrier (BBB): no detectable rutin was observed in brain tissue 12 h following intravenous administration of free rutin. Upon entering systemic circulation, parent rutin undergoes rapid hepatic first-pass metabolism and predominantly accumulates in peripheral organs including the liver and lungs, limiting its access to brain or intracranial tumor sites. Unlike rutin, intraperitoneally administered quercetin aglycone (10, 30, 50 mg/kg) has been shown to alleviate blood–brain barrier disruption and reduce vascular permeability in cerebral ischemia–reperfusion injury preclinical models. Current evidence does not support efficient blood–brain barrier (BBB) penetration of intact rutin; instead, quercetin aglycone and certain metabolites are reported to be more likely to reach the central nervous system in preclinical models [25,26]. Quercetin and its conjugated metabolites represent the dominant circulating species following oral administration and are generally considered the primary mediators of systemic biological effects. These metabolites have been widely reported to exert antioxidant, anti-inflammatory, and vasoprotective activities, which may contribute to their potential roles in chronic systemic conditions such as cardiovascular diseases, metabolic disorders, and vascular dysfunction [27]. However, the relative contributions of individual conjugated forms remain incompletely understood. In addition, unabsorbed rutin and quercetin can undergo further microbial metabolism in the colon, generating smaller phenolic compounds such as protocatechuic acid and other derivatives. These microbial phenolic metabolites may exert local effects within the gastrointestinal tract, including modulation of gut microbiota, anti-inflammatory activity, and maintenance of intestinal barrier function, suggesting potential relevance in intestinal disorders [28,29].

### 3.5. Effects of Gut Microbiota on the Metabolism of Rutin

The biological effects of rutin may depend to a large extent on its activation efficiency within the gut, which can be conceptualized as a natural prodrug system. From a pharmacomicrobiomics perspective, rutin metabolism is not a simple linear process but rather a multi-branch pathway exhibiting high interindividual heterogeneity. The rate-limiting step begins with specific enzymes secreted by the gut microbiota, particularly α-L-rhamnosidase, whose activity is a major determinant of the conversion rate of rutin to quercetin [30]. Studies have found that abundant bacterial species such as *Parabacteroides distasonis* and *Enterococcus casseliflavus* can rapidly release the active moiety, whereas individuals lacking such specific enzymes, for example those with a Lactobacillus-predominant gut environment, show marked metabolic lag, leading to accumulation of intact rutin in the lower intestine [31]. A deeper metabolic divergence occurs at the stage of C-ring cleavage following quercetin generation. The core gut microbiota metabotypes of different individuals may influence the chemical nature of the degradation products. Under the catalysis of *Eubacterium ramulus* or *Flavonifractor plautii*, quercetin is primarily converted to 3,4-dihydroxyphenylacetic acid (DOPAC). Transcriptomic profiling confirms that differential expression of pirin-like protein governs this pathway divergence. In vitro clonogenic assays demonstrate that DOPAC exerts anti-proliferative activity against HCT116 colon cancer cells at concentrations above 15.62 μM, and the culture supernatant of *F. plautii* achieves around 65% colony inhibition. Other gut flora mainly produces short-lived protocatechuic acid with strong antioxidant properties. Such metabolic discrepancies mean identical oral rutin doses may trigger divergent biological outputs across populations, ranging from central neuroprotective potential to peripheral anti-inflammatory effects. Preliminary cellular models preliminarily indicate DOPAC may cross the blood–brain barrier and confer neuroprotective activity, yet quantitative in vivo brain distribution data remain insufficient for definitive validation [32]. Under alternative microbial backgrounds, metabolism tends to produce protocatechuic acid [33], which has strong antioxidant activity but an extremely short half-life. This qualitative difference in metabolites may help explain why identical doses of rutin could be associated with distinctly different patterns of biological activity, such as neuroprotection versus systemic anti-inflammatory effects, across different individuals.

This qualitative and quantitative variability in microbial metabolism could contribute to substantial interindividual differences in therapeutic responses to rutin. It also highlights an importance of conventional rutin formulation development: strategies that do not account for gut microbiota variability may limit therapeutic reproducibility across diverse patient populations. This hypothesis, however, requires further clinical validation, as direct evidence of microbiota-dependent efficacy differences in humans remains limited.

## 4. Effects of Rutin Metabolism on Pharmacological Activity

### 4.1. Alteration of Bioactive Components

Although rutin itself exhibits antioxidant and anti-inflammatory activities in in vitro assays, systemic in vivo effects appear to be largely attributable to the contribution of its metabolites. Studies have shown that many rutin metabolites, such as quercetin and its glucuronidated derivatives, display comparable or even superior bioactivity to the parent compound [34]. Notably, colonic microbial metabolites also contribute to tissue-specific effects. For instance, rutin is rapidly degraded by rumen microorganisms in dairy cows, yielding 4-methylcatechol sulfate, which exerts antioxidant, anti-inflammatory, vasodilatory, and antiplatelet effects. This metabolic conversion simultaneously blocks the formation of the harmful substance p-cresol from tyrosine, thereby reducing toxin accumulation and odor emissions in the body [35]. This example further supports the view that systemic bioactive species following oral administration are predominantly quercetin-derived metabolites, although parent rutin may contribute to local gastrointestinal effects.

### 4.2. Regulatory Targets

Given that the parent compound shows very limited access to systemic target tissues after oral dosing, many of rutin’s pharmacological effects are indirectly mediated by its metabolites. For example, Zhang [36] reported that quercetin only weakly inhibits α-glucosidase (IC_50_ = 24.55 μM) and exhibits no inhibitory activity against fructose-1,6-bisphosphatase (FBPase), resulting in modest hypoglycemic and hypolipidemic effects. In contrast, its derivative 8-prenylquercetin potently inhibits both α-glucosidase (IC_50_ = 4.38 μM, approximately 75-fold more potent than acarbose) and FBPase (IC_50_ = 3.62 μM, comparable to AMP). Furthermore, it activates the AMPK/ACC/CPT-1A signaling pathway, promotes cellular glucose consumption, and reduces lipid accumulation, demonstrating superior multi-target synergistic hypoglycemic and hypolipidemic effects.

### 4.3. Improved Pharmacokinetics

Metabolic transformation substantially changes the pharmacokinetic behavior of rutin. Following conversion to quercetin and its conjugates, however, absorption is significantly improved. Moreover, the increased water solubility of phase II conjugates facilitates their distribution to peripheral tissues via systemic circulation.

Researchers characterized the pharmacokinetic profile of rutin-derived metabolites in humans [37]. In this study, healthy volunteers received 500 mg of rutin daily. The major circulating species identified were quercetin-3-O-glucuronide, isorhamnetin, and kaempferol, with no intact rutin detected in plasma. The maximum plasma concentration (C_max_) of quercetin metabolites ranged from 0.04 to 0.22 μmol/L, and the time to maximum concentration (T_max_) ranged from 4 to 7 h. Notably, two of the three subjects reached peak concentrations at 7 h, while one peaked at 4 h, reflecting interindividual variability in the rate of microbial hydrolysis. Complete elimination was not achieved within the 24 h sampling period, consistent with the prolonged residence time conferred by enterohepatic recycling of conjugated metabolites. The metabolites were primarily excreted via urine. The conjugating enzymes involved in quercetin conjugation were identified as UDP-glucuronosyltransferases (UGTs) and catechol-*O*-methyltransferase (COMT).

Researchers quantitatively characterized the kinetics of quercetin phase II metabolism at the cellular level using an HEK293 cell model overexpressing sulfotransferase 1A3 (SULT1A3). At a quercetin concentration of 5 μM, the major product was quercetin-*O*-sulfate, predominantly the 3′-*O*-sulfate isomer. The enzymatic kinetic parameters were determined as follows: K_m_ of 6.00 ± 1.41 μM, V_max_ of 1.74 ± 0.14 nmol·min^−1^·mg^−1^, and intrinsic clearance (CLint) of 0.29 ± 0.07 mL·min^−1^·mg^−1^. Notably, the fraction of quercetin metabolized could be reduced by up to 76.9% through pharmacological inhibition of the efflux transporters breast cancer resistance protein (BCRP) and multidrug resistance-associated protein 4 (MRP4), indicating that cellular sulfation is tightly coupled to transporter-mediated efflux. The regulatory enzyme arylsulfatase B was also identified as a modulator of intracellular quercetin sulfate disposition.

Collectively, these pharmacokinetic data suggest that the systemic exposure to rutin-derived active species is governed not by the absorption of the parent compound, but by the sequential processes of microbial deglycosylation, phase II conjugation, and transporter-mediated cellular efflux. The substantial interindividual variability in T_max_ highlights the critical role of gut microbiota composition in determining the rate and efficiency of rutin metabolic activation. These findings support the importance of metabolite-resolved pharmacokinetic endpoints in evaluating rutin formulations and provide a mechanistic foundation for the ADME-regulatory classification framework proposed in this review.

### 4.4. Individual Differences

Although gut microbiota heterogeneity is the primary factor driving variability in rutin metabolism host genetic polymorphisms, physiological characteristics and pathological states also play nonnegligible roles in determining its final bioavailability.

First, genetic polymorphisms constitute the molecular basis of interindividual differences. Quercetin generated from rutin hydrolysis is primarily conjugated by phase II metabolizing enzymes in the small intestine and liver, such as UDP glucuronosyltransferases (UGTs) UGT1A1 and UGT1A9, and catechol O methyltransferase (COMT). Studies have shown that promoter polymorphisms in UGT genes can significantly alter the composition ratio and excretion rate of conjugated metabolites in the blood [38]. Furthermore, genetic differences in the expression levels of flavonoid transporters, such as sodium–glucose-linked transporter 1 (SGLT1) and multidrug resistance-associated protein 2 (MRP2), may contribute to why the C_max_ often varies several fold among different subjects receiving the same dose [39].

Second, host pathophysiological states dynamically reshape the absorption kinetics of rutin. In pathological models such as inflammatory bowel disease (IBD) or diabetes, although increased intestinal barrier permeability, often referred to as leaky gut, may enhance passive diffusion of intact rutin, the accompanying downregulation of transporters and alterations in mucosal blood flow tend to reduce active transport efficiency [40]. Meanwhile, interindividual differences in gastric emptying determine the distribution of residence time of rutin between the proximal small intestine, the primary absorption site, and the distal large intestine, the site of microbial conversion, thereby altering the in vivo exposure ratio of the parent drug to its metabolites [41].

Finally, demographic factors, including age and sex, further add to the complexity of clinical translation. Reduced gastric acid secretion and altered gut microenvironment in the elderly often slow the dissolution rate of solid rutin formulations. Due to inherent sex differences in the abundance of phase II metabolizing enzymes, females exhibit longer circulating half-lives of flavonoids than males in some studies [42]. This multidimensional heterogeneity suggests that precision nutrition and precision therapy based on gut microbiota characterization and host genotype represent promising directions to address the bottleneck of rutin clinical translation. Future formulation development should consider this heterogeneity to achieve personalized efficacy optimization.

### 4.5. Summary

Finally, these findings offer the following implications for the development and application of rutin-based formulations. First, viewing rutin from a prodrug perspective helps explain the long-standing paradox of low bioavailability yet high bioactivity, emphasizing that efficacy evaluation should incorporate circulating metabolites in addition to the intact parent compound. Second, the in vivo metabolic trajectory of rutin provides a theoretical basis for the rational design of efficacy-enhancement strategies. The core challenge shifts from passively overcoming poor solubility and permeability to actively controlling the metabolic fate of rutin in accordance with specific therapeutic goals. Third, the substantial multi-dimensional heterogeneity in rutin metabolism and pharmacokinetics highlights the necessity of personalized applications. A uniform enhancement strategy may not be optimal for all populations. Future development of rutin-based functional foods or pharmaceuticals should be established within a framework of precision dosing, based on thorough assessment of individual physiological backgrounds including gut microbiota composition, genetic characteristics, pathological state, age, and sex.

## 5. Strategies for Enhancing the Efficacy of Rutin

As described in previous chapters, oral rutin undergoes gut microbial deglycosylation followed by phase II conjugation to form circulating quercetin derivatives. This metabolic feature shifts formulation goals from simple solubility improvement to targeted control of active metabolite generation, matching distinct therapeutic demands.

Two major categories of enhancement strategies have been intensively explored: nanotechnology-based delivery systems [43] and structural modification [44]. Unlike previous reviews that primarily list these approaches qualitatively, the present section aims to: (i) innovatively classify nanodelivery systems according to their metabolic regulation objectives; (ii) systematically compare the two core strategies of nanodelivery and structural modification across multiple dimensions including pharmacokinetics, metabolic regulation, safety, and translational feasibility; and (iii) establish an actionable metabolism-guided decision framework to provide a theoretical basis for the rational design of oral rutin formulations.

### 5.1. Nano-Delivery Strategies for Oral Rutin: An ADME-Regulatory Framework

Conventional classifications of rutin nanoformulations are based on carrier materials, such as liposomes, polymeric nanoparticles, nanoemulsions, metallic nanoparticles, and solid lipid nanoparticles [43]. These classifications are useful from a pharmaceutical engineering perspective. However, they do not explain how nanocarriers regulate the in vivo fate of orally administered rutin. Oral rutin rarely exists as intact glycoside in systemic circulation; microbial transformation generates quercetin glucuronide, sulfate and methyl conjugates as major bioactive moieties. Therefore, the central challenge in rutin formulation is not simply increasing parent compound exposure. It is controlling where, when, and in what molecular form active species are generated.

**Figure 2:**
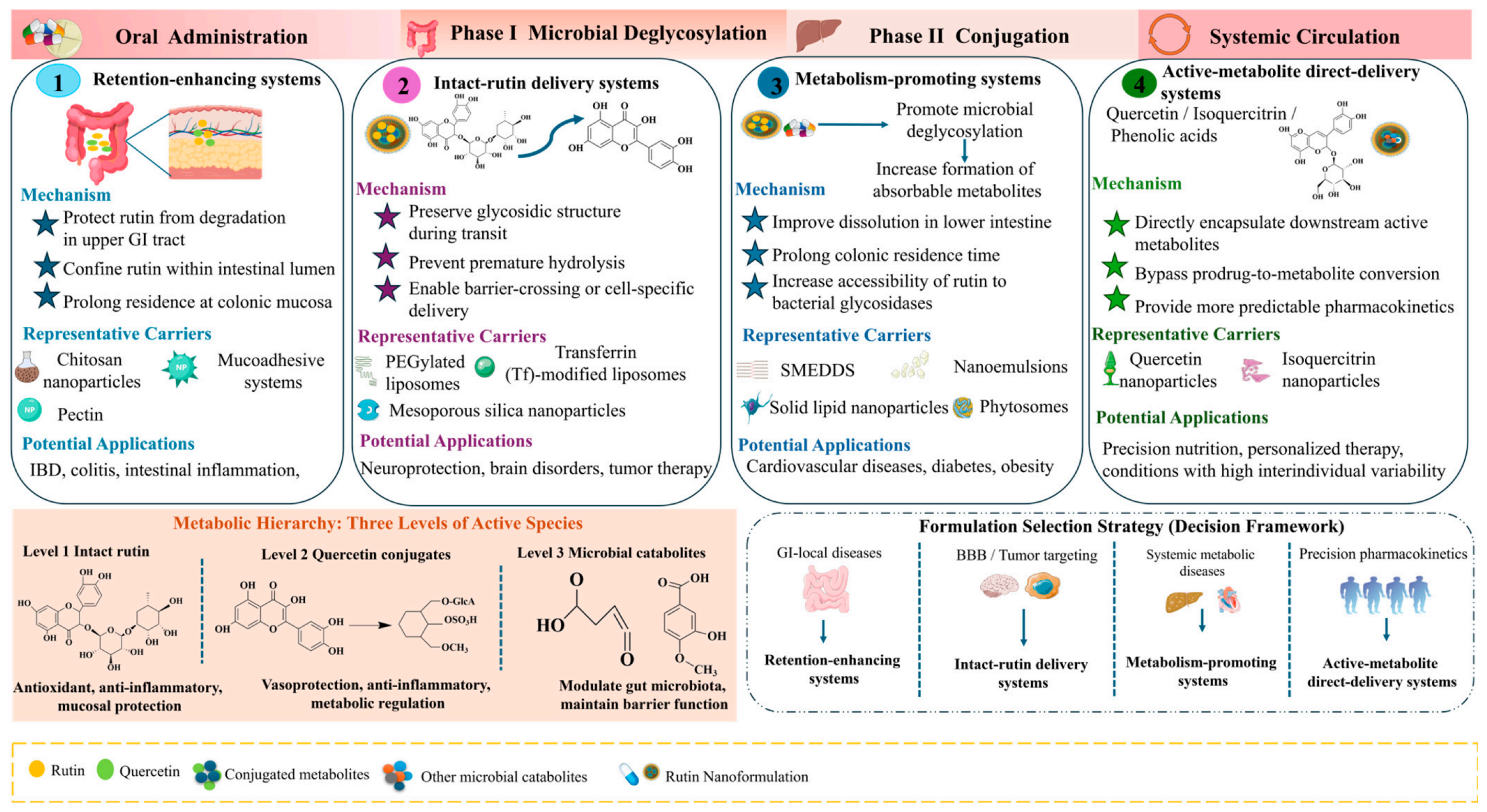
ADME-oriented functional classification of rutin nano-delivery systems.

Based on this understanding, we propose an ADME regulatory classification framework. This framework categorizes nano-delivery systems according to their primary pharmacokinetic objective, rather than carrier composition. Four functional classes are defined, reflecting increasing intervention along the rutin metabolic pathway. (i) Retention-enhancing systems are designed to confine intact rutin within the intestinal lumen and prolong its residence time at the colonic mucosa. These systems protect rutin from premature degradation in the upper gastrointestinal tract and enhance local antioxidant and anti-inflammatory activities within the intestinal microenvironment. Representative mucoadhesive and colon-retentive formulations based on chitosan and related polymers have demonstrated prolonged mucosal retention and improved local pharmacodynamic activity in intestinal disease models [45]; (ii) Intact-rutin delivery systems aim to preserve the glycosidic structure of rutin during gastrointestinal transit and systemic circulation, thereby enabling the parent compound to reach distal target tissues. This strategy is particularly relevant for tissues protected by biological barriers, where targeted nanocarriers may facilitate tissue-specific delivery of intact rutin prior to local activation or intracellular uptake. Representative ligand-modified liposomes and targeted nanocarriers have shown enhanced tissue accumulation and improved pharmacokinetic behavior in preclinical studies [46]; (iii) Metabolism-promoting systems are based on the recognition that quercetin and its phase II conjugates are the major circulating bioactive species following oral rutin administration. These systems facilitate microbial deglycosylation and metabolite generation by improving dissolution in the lower intestine, prolonging colonic residence, and increasing the accessibility of rutin to bacterial glycosidases. Several nanoformulations have demonstrated enhanced systemic exposure to quercetin glucuronides and sulfates in vivo through improved microbiota-mediated biotransformation [47]; (iv) Active-metabolite direct-delivery systems represent the most direct strategy for minimizing interindividual variability associated with gut microbiota composition and host metabolic capacity. Instead of relying on in vivo bioactivation of rutin, these systems directly encapsulate downstream active metabolites such as quercetin, isoquercitrin, or specific phenolic acids. Representative metabolite-loaded nanoformulations have demonstrated more predictable pharmacokinetic behavior and reduced dependence on microbiota-mediated activation pathways. However, this strategy may sacrifice the sustained-release characteristics associated with gradual microbial hydrolysis of native rutin [48]. The conceptual framework and corresponding intervention sites along the oral ADME pathway of rutin are illustrated in Figure 2.

Representative studies classified within this framework are summarized in Table 1. It should be noted that the majority of published nanoformulations fall into the intact-rutin delivery category. Studies explicitly designed as retention-enhancing, metabolism-promoting, or metabolite-direct systems remain scarce. This imbalance reflects the historical focus on systemic delivery rather than on metabolic fate regulation.

The translational status of major nano-delivery systems is further evaluated in Table 2. This evaluation considers ADME regulatory objectives, the identity of circulating species, pharmacokinetic improvements, human relevance, and clinical maturity.

To improve the translational significance of the comparative analysis, the human relevance of each formulation strategy was qualitatively assessed according to its ability to address clinically relevant oral ADME processes. The evaluation primarily considered whether the study investigated metabolite-resolved pharmacokinetics, particularly quercetin glucuronides, sulfates, and methylated conjugates; whether gut microbiota-mediated metabolism and enterohepatic circulation were considered; whether systemic exposure, tissue distribution, and oral pharmacokinetic behavior were evaluated using clinically relevant in vivo models; and whether the formulation demonstrated translational feasibility, including safety, scalability, reproducibility, and potential regulatory applicability. A structured scoring matrix with seven evaluation dimensions is provided in Table S1 (Supplementary Materials) to ensure transparency and reproducibility of the classification. Detailed scoring records for rutin nano-delivery systems corresponding to the main text are summarized in Table S2 (Supplementary Materials).

Importantly, the present review defines human relevance as translational relevance rather than pharmacological efficacy alone. Therefore, formulations evaluated only through in vitro release behavior, particle characterization, or parent-rutin exposure in rodents were considered to have limited human relevance, because these endpoints do not adequately reflect the actual circulating bioactive species following oral rutin administration. In contrast, studies incorporating metabolite-resolved pharmacokinetics, clinically relevant ADME evaluation, or translational evidence were considered to possess relatively higher human relevance.

Among the evaluated systems, transferrin-modified brain-targeted liposomes represent the most extensively characterized formulation. They achieved a 2.77-fold increase in brain exposure in rats. However, this finding has not been validated in humans. It also remains unknown whether the observed effects are mediated by intact rutin or by intracellularly generated metabolites. Nanoemulsions and nanosuspensions enhance dissolution and permeability and likely increase systemic exposure to quercetin conjugates rather than intact rutin. However, direct quantitative evidence for this inference is lacking.

Despite the diversity of strategies, clinical translation of rutin nanoformulations remains constrained by three major bottlenecks. First, pharmacokinetic characterization is incomplete. Most studies lack metabolite-resolved PK parameters such as absolute oral bioavailability and tissue distribution. This makes cross-study comparisons difficult. Second, clinical evidence is entirely absent. All reported systems are at the preclinical stage, and no human pharmacokinetic or safety data exist. Third, scalable manufacturing and long-term safety remain unresolved. Key challenges include batch-to-batch consistency, storage stability, and sterilization methods. For metal-based carriers, the risks of chronic toxicity and tissue accumulation require systematic evaluation.

These bottlenecks indicate that future research should prioritize three directions. First, metabolite-resolved pharmacokinetic profiling is needed to clarify which molecular species drive therapeutic effects. Second, studies should advance from rodent models to larger animals with human-relevant physiology. Third, scalable and cost-effective manufacturing processes suitable for industrial production must be developed. Only by addressing these gaps can the clinical potential of rutin nano-delivery systems be rigorously assessed.

### 5.2. Structural Modification

Structural modification aims to alter rutin’s intrinsic physicochemical properties at the molecular level, generating derivatives with improved solubility, permeability, or bioactivity, while sometimes retaining the advantage of being a single chemical entity with a defined regulatory path [63]. Table 3 summarizes several representative studies concerning the structural modification of rutin.

As shown in Table 3, glycosylation, metal complexation, esterification, hydroxyethylation, and benzylation are the main strategies for the structural modification of rutin. Structural modification reprograms the pharmacokinetic trajectory of rutin by directly altering its physicochemical properties at the molecular level. Certain modifications, such as glycosylation, primarily accelerate the dissolution step without fundamentally altering metabolic dependence on the gut microbiota. For instance, González-Alfonso [71] demonstrated that polyglucosylation of rutin catalyzed by cyclodextrin glucanotransferase achieved a 60% conversion yield and generated maltooligosyl derivatives with substantially enhanced aqueous solubility. Kadota [72] further elucidated that α-glucosyl rutin (Rutin-G) enhances the dissolution of hydrophobic flavonoids through the formation of polydisperse aggregates, providing a molecular mechanism for the solubilizing effect. Other modifications, such as hydroxyethylation, partially uncouple absorption from microbial hydrolysis, thereby altering metabolic fate itself. Troxerutin, the hydroxyethylated derivative of rutin, represents the only clinically validated example of this strategy and has demonstrated improved bioavailability and well-characterized pharmacokinetic profiles [73]. Esterification and benzylation shift partition coefficients toward higher lipophilicity, potentially redirecting absorption from the paracellular to the transcellular route or enabling transdermal administration. Mexia [66] reported that enzymatic esterification of rutin using Candida antarctica lipase B increased lipophilicity by approximately 36.5-fold without compromising antioxidant activity. da Silva [69] synthesized a benzylated rutin derivative (RuDiOBn) that exhibited a 42.3-fold increase in lipophilicity and a 6.1-fold increase in skin permeability, with no cytotoxicity toward human fibroblasts. Metal complexation can generate entirely new pharmacological activities absent in the parent compound. Wu [74] reported that flavonol-Ru(II) complexes, representing the first report of free radical scavenging abilities for such complexes, exhibited potent antioxidant and anticancer activities, with one complex demonstrating efficacy comparable to 5-FU against A549 non-small-cell lung cancer cells. These distinctions in ADME regulatory mechanisms are critical for matching structural modification strategies to specific therapeutic goals.

Beyond the types listed above, other strategies such as etherification, radioactive labeling, and polymer conjugation have also been reported for rutin modification [75,76,77]. However, studies on these strategies are mostly at an early exploratory stage, lacking systematic pharmacokinetic and clinical translation data, and are therefore not detailed in this review.

Current non-troxerutin structural derivatives share three core deficiencies: insufficient in vivo pharmacokinetic characterization, ambiguous metabolic pathways, and complete absence of human clinical data. Future structural optimization should prioritize reducing reliance on intestinal flora, conducting full ADME profiling, and targeting diseases with definite clinical demands as troxerutin does.

### 5.3. Successful Case of Structural Modification: Troxerutin

Among all rutin derivatives summarized in Table 3, troxerutin (hydroxyethylrutosides) is the only commercially marketed venoprotective agent with abundant standardized human pharmacokinetic and clinical trial evidence, which fundamentally distinguishes it from other in vitro/animal-only rutin formulations. Beyond its well-established vascular protective activity, preclinical studies have confirmed that troxerutin also possesses neuroprotective and analgesic properties, as well as therapeutic effects for hemorrhoid treatment, broadening its pharmacological application potential [78,79,80,81,82]. As systematically reviewed by Wadworth and Faulds [83], troxerutin is a semi-synthetic flavonoid mixture officially approved by drug authorities across Europe, China and other regions under the brand name Venoruton, with standardized oral tablet/capsule formulations for decades of clinical application [84]. Its human pharmacokinetic characteristics have been fully characterized in healthy volunteer trials: the oral bioavailability reaches approximately 10%. Part of troxerutin can be directly absorbed in the gastrointestinal tract without complete deglycosylation by intestinal flora, while unabsorbed fractions are degraded into aglycone metabolites by gut microbes. Mono-, di- and tri-hydroxyethylrutoside components undergo hepatic metabolism to form glucuronide conjugates, whereas the more polar tetra-hydroxyethylrutoside is mainly eliminated via the kidney. After oral administration, the peak plasma concentration reaches within 1–6 h, and the elimination half-life ranges from 10 to 25 h, owing to enterohepatic circulation; only 3–6% of the administered dose is excreted through urine, with bile serving as the primary excretion pathway. A large number of randomized, double-blind, placebo-controlled human trials have verified its therapeutic efficacy for chronic venous insufficiency (CVI): daily oral doses of 0.9–1.2 g significantly alleviate leg swelling, pain, night cramps and heavy limb sensation, with 73–100% of patients achieving symptomatic improvement, superior to placebo groups. It also shows definite clinical benefits for gestational varicosis, severe hemorrhoids and diabetic microangiopathy without interfering with glycemic control [84,85,86]. Long-term post-marketing safety monitoring confirms that troxerutin is well tolerated, with only mild transient gastrointestinal adverse reactions, and no drug–drug interaction with anticoagulants such as warfarin [83]. Furthermore, industrialized mature semi-synthetic production technology enables large-scale commercial supply, rather than limited laboratory preparation. In vivo animal studies further support its venotonic, anti-inflammatory and anti-angiogenic mechanisms by regulating VEGF/eNOS and inflammatory cytokines TNF-α, IL-1β [87]. In stark contrast, all other rutin structural modifications and nano-delivery strategies listed in Table 2 and Table 3 merely rely on cellular or rodent animal data, lacking human pharmacokinetic profiles, formal clinical trials and official marketing approval, which explains why troxerutin is categorized as “marketed compound” and assigned the highest human relevance grade (High) in our scoring system. Complete detailed scoring results are available in Table S3 (Supplementary Materials).

### 5.4. Other Formulation Strategies

Beyond nanotechnology and structural modification, formulation techniques such as cyclodextrin inclusion complexes, solid dispersions, and phospholipid complexes have also been employed to improve the solubility and bioavailability of rutin [88,89,90]. The above strategies fall under the category of formulation technologies, similar to nanotechnology, but they typically do not involve active targeting or intervention in the absorption pathway. Compared with structural modification, they do not generate new chemical entities and therefore do not require comprehensive toxicological evaluation as new drugs, offering a lower technical threshold for functional food and nutraceutical development. However, most relevant research merely evaluates in vitro dissolution and animal absorption, lacking systematic ADME and clinical validation data.

### 5.5. Quantitative Comparison and Strategy Selection

#### 5.5.1. Comprehensive Comparative Analysis

Based on the systematic overview of nanotechnology-based delivery and structural modification strategies for rutin, Table 4 presents a multi-dimensional comparison of the two approaches, covering their core mechanisms of action on rutin’s in vivo ADME and metabolic fate, representative improvements in oral bioavailability, tissue-targeting capability, regulatory status, and clinical translational potential.

First, the two strategies differ fundamentally in their effect on metabolic fate. Nanotechnology can actively intervene in rutin’s in vivo metabolic pathway through carrier design: for instance, encapsulation in lipid-based carriers can bypass the colonic environment, avoiding premature microbial hydrolysis [91], while nanoemulsion can achieve site-specific release in the colon, preserving the glycoside structure for local effects [92]. In contrast, structural modification generally does not alter rutin’s dependence on gut microbiota; troxerutin is the only known derivative that can be absorbed without complete deglycosylation. This distinction has direct implications for therapeutic applications. When interindividual variability in gut microbiota composition must be minimized, strategies that bypass or actively enhance microbial metabolism, such as intact-rutin nano-delivery or metabolism-promoting nano-systems, may be preferred. When systemic exposure to quercetin conjugates is the therapeutic goal and the patient population has adequate microbial function, structural modification offers a more practical route.

Second, the targeting capabilities and implementation approaches of the two strategies differ significantly. Unmodified nanocarriers can achieve passive targeting through the enhanced permeability and retention effect in tumor tissues, enabling the accumulation of drugs at lesion sites without additional ligand modification. When conjugated with specific ligands such as folic acid or transferrin, nanocarriers can further achieve active targeting. For instance, folic acid-functionalized carbon dots loaded with rutin can specifically bind to folate receptors overexpressed on MCF-7 breast cancer cells, facilitating receptor-mediated internalization and precise drug delivery to tumor cells [93]. In contrast, structural modifications of rutin itself that merely optimize physicochemical properties, including glycosylation to improve water solubility, hydroxyethylation to enhance bioavailability, and acylation to increase lipophilicity, do not confer targeting capability. Such modified rutin derivatives still require a nanocarrier platform for targeted delivery. It is important to note, however, that when structural modification involves the direct conjugation of targeting ligands to the rutin molecule, the resulting derivative can possess active targeting ability on its own, eliminating the need for additional carriers.

Third, translational readiness differs markedly. Troxerutin, as a successful example of structural modification, has been on the market for years with robust clinical evidence. In contrast, despite promising results in animal studies, no rutin nanoformulation has yet entered clinical trials, facing translational barriers such as scalability and safety evaluation.

#### 5.5.2. Strategy Selection

Based on the above quantitative comparison, in practical applications, it is necessary to select the most appropriate efficiency improvement strategy according to different application scenarios.

Situations favoring nanotechnology primarily include the following: therapies demanding intact rutin delivery to target sites, as carriers shield rutin from intestinal microbial hydrolysis; tumor-targeted treatment relying on EPR passive effect or ligand-mediated active delivery; controlled spatiotemporal release requirements; co-delivery of multiple active ingredients; and interventions aiming to reduce microbiota-induced interindividual efficacy fluctuations.

**Figure 3:**
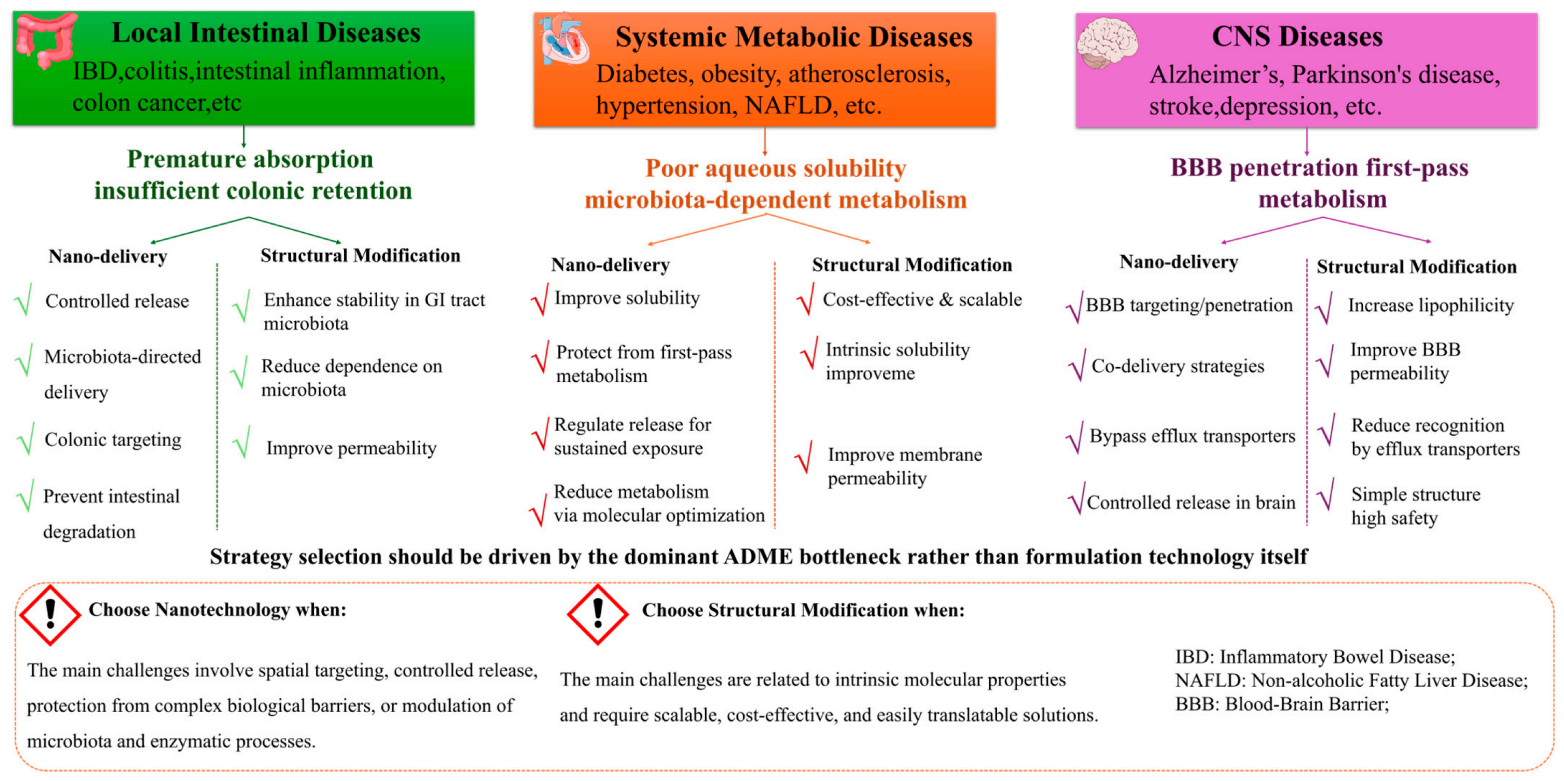
Schematic strategy selection framework for improving the oral bioavailability of rutin.

Situations favoring structural modification primarily include the following. When quercetin and its conjugates are acceptable as the active moieties, enhancing solubility and absorption through glycosylation or hydroxyethylation represents a direct strategy, as exemplified by the success of troxerutin. For local or topical applications such as skin care and wound healing, the benzylated derivative RuDiOBn exhibits favorable skin permeability, allowing structural modification to be tailored to specific administration routes. When development of a well-defined single chemical entity is sought, structural modification yields new chemical entities with a relatively clear regulatory pathway, as seen with troxerutin. Moreover, modified derivatives such as α-glucosyl rutin have already found applications in cosmetics and functional foods. The detailed strategy selection process is shown in Figure 3.

This framework is based on the available evidence, which is unevenly distributed and incomplete in several important respects. Metabolite-resolved pharmacokinetic data are absent for most formulations and derivatives. The relative pharmacological contributions of individual circulating species remain poorly defined. Human clinical evidence is limited to troxerutin for chronic venous insufficiency. As these evidence gaps are addressed, the decision framework should be refined with more precise quantitative thresholds and expanded to incorporate patient-specific factors such as gut microbiota composition and host metabolic capacity.

#### 5.5.3. Prospects for Synergistic Application

Although nanotechnology and structural modification each offer unique advantages, both strategies have their own limitations. The major challenges for nanotechnology lie in manufacturing complexity, batch-to-batch consistency, and the need for systematic safety evaluation of carrier materials, with no clinical translation achieved to date. For structural modification, the primary challenges include the requirement for comprehensive toxicological evaluation for each new chemical entity, and the fact that most derivatives still depend on microbial metabolism, leaving the issue of interindividual variability unresolved.

The limitations of the two strategies are complementary, providing a rationale for synergistic approaches. The most advanced strategy combines both: encapsulating a structurally optimized derivative into a functionalized nanocarrier to achieve both molecular optimization and precision delivery. For instance, troxerutin, a hydroxyethylated derivative of rutin with structurally enhanced water solubility, has been encapsulated into solid lipid nanoparticles (TXR-SLNs) via high-shear homogenization combined with ultrasonication. The optimized formulation, designated TXR-SLN2, exhibits a uniform particle size of 140.5 ± 1.02 nm and an entrapment efficiency of 83.62%, and achieves sustained drug release with a cumulative release of 82.47% at 24 h, effectively addressing troxerutin’s remaining limitations of rapid plasma clearance and poor membrane penetration [94]. This example demonstrates that nanotechnology serves as the core synergistic pathway for rutin and its derivatives. Structural modification optimizes the molecular properties of rutin, while nanocarriers compensate for the derivative’s delivery defects. Although still in early exploration, this dual-optimization approach represents a promising direction for future development.

#### 5.5.4. Conclusions

This section provides a quantitative comparison between nanotechnology and structural modification, revealing fundamental differences in their effects on metabolic fate, targeting capability, and translational readiness. Based on this analysis, a decision framework is proposed: nanotechnology is preferred when protection of intact rutin, targeted delivery, or circumvention of interindividual variability is required; structural modification is more suitable when metabolites are acceptable as active species, topical applications are intended, or a well-defined chemical entity is sought. The limitations of the two strategies are complementary, and their synergistic application holds promise for achieving superior therapeutic outcomes, representing a future direction for development.

### 5.6. Regulatory Status of Rutin

In the European Union, health claims for food constituents are regulated under Regulation (EC) No 1924/2006. In 2010, the EFSA Panel on Dietetic Products, Nutrition and Allergies (NDA) evaluated two health claims submitted for rutin: improvement of endothelium-dependent vasodilation (ID 1649, 1783) and protection of DNA, proteins and lipids from oxidative damage (ID 1784). Characterisation of rutin: The EFSA Panel considered that rutin is present in many plants including citrus fruits, berries, apples, and buckwheat, can be analysed by HPLC methods, and is cleaved in the intestine to quercetin, which is absorbed and metabolised in the human organism. The Panel concluded that rutin is sufficiently characterised for the evaluation of health claims.

Regarding improvement of endothelium-dependent vasodilation, the claimed effect referred to vascular health and circulatory system, with the general population as the target. The Panel considered that improvement of endothelium-dependent vasodilation may be a beneficial physiological effect. However, after reviewing the four references provided, the Panel stated that no conclusions could be drawn from these references for the scientific substantiation of the claimed effect. Consequently, the Panel concluded that a cause and effect relationship has not been established between the consumption of rutin and the improvement of endothelium-dependent vasodilation.

Regarding protection of DNA, proteins and lipids from oxidative damage, the claimed effect referred to antioxidant properties, with the general population as the target. The Panel considered that protection of DNA, proteins and lipids from oxidative damage may be a beneficial physiological effect. After reviewing the three references provided, the Panel focused on one randomised, single-blind, placebo-controlled human intervention study in which 18 healthy female volunteers consumed 500 mg/day of rutin or placebo for six weeks. Various markers were measured, including plasma antioxidant capacity, malondialdehyde (MDA) content, lymphocyte DNA damage, urinary 8-hydroxy-2-deoxyguanosine, and urinary 8-iso-prostaglandin F2α. The Panel noted that the only human intervention study provided did not support an effect of rutin consumption on the protection of DNA, proteins or lipids from oxidative damage. Therefore, the Panel concluded that a cause and effect relationship has not been established between the consumption of rutin and the protection of DNA, proteins and lipids from oxidative damage [95].

The EFSA conclusions reflect a core translational bottleneck for rutin: despite extensive in vitro and animal studies suggesting multiple pharmacological activities, high-quality human evidence remains severely lacking. Notably, the EFSA Panel explicitly acknowledged that rutin is cleaved in the intestine to quercetin and absorbed. However, even this well-characterised metabolic feature did not compensate for the absence of demonstrable effects in human intervention studies. This regulatory status has important implications for product development: for nutraceutical and functional food applications, manufacturers must avoid making unauthorised health claims, and for pharmaceutical development, rigorous clinical trials are required to establish efficacy, regardless of the compound’s natural origin. Only when the pharmacokinetic profile of rutin is substantially improved can consistent and measurable clinical effects be expected in human trials.

Beyond the lack of clinical evidence, uncertainties regarding rutin’s genotoxic potential also contribute to regulatory caution. Sanz-Serrano [96] conducted a comprehensive in vitro assessment following EFSA guidelines, demonstrating that rutin exhibits direct frameshift mutagenicity in the Ames test independent of metabolic activation, while micronucleus test results were equivocal due to high cytotoxicity, and no DNA strand breaks or oxidative damage were detected in the comet assay. Notably, human plasma concentrations of rutin are 500–5000 times lower than the genotoxic threshold, but local colonic exposure risks remain to be clarified.

## 6. Limitations of the Present Review

This work is a narrative review rather than a systematic review with predefined registration protocol and standardized literature retrieval criteria. Several inherent limitations should be acknowledged. First, the selection of included publications relies on subjective screening, and strict quantitative literature quality evaluation and risk-of-bias assessment were not performed. Second, considerable heterogeneity exists among the collected original studies, including differences in rutin sources, modification routes, formulation preparation parameters, dosing regimens, animal species, detection indicators and evaluation durations. Such discrepancies lead to insufficient direct comparability of experimental outcomes across independent investigations. Third, most available data are derived from in vitro cell tests and rodent preclinical experiments; high-quality human pharmacokinetic research and randomized controlled clinical trials remain scarce for nearly all novel rutin derivatives and nanoformulations. Apart from troxerutin, translational evidence supporting clinical application is inadequate. Lastly, grey literature, unpublished raw data and studies written in non-English languages were not comprehensively retrieved, which may cause potential publication bias.

## 7. Summary and Discussion

This review redefines the development strategy of oral rutin formulations from a metabolism-centered perspective. Rather than functioning as a conventional orally absorbed flavonoid, rutin primarily acts as a microbiota-activated prodrug. Following oral administration, rutin undergoes extensive deglycosylation and phase II conjugation, generating quercetin glucuronides, sulfates, methylated metabolites, and downstream phenolic acids that constitute the major bioactive species in vivo. Therefore, the key challenge in rutin formulation is not simply improving parent-compound bioavailability, but regulating where, when, and in what molecular form active metabolites are generated. Based on this concept, an ADME-oriented analytical framework is proposed to systematically connect metabolic fate with formulation strategy selection. Rutin nano-delivery systems are functionally reclassified into retention-enhancing systems, intact-rutin delivery systems, metabolism-promoting systems, and active-metabolite direct-delivery systems according to their primary pharmacokinetic objectives. Different systems may therefore be more suitable for distinct therapeutic purposes, including intestinal disorders, systemic metabolic diseases, or tissue-targeted applications. This review further compares nanotechnology and structural modification from the perspectives of pharmacokinetics, metabolic regulation, translational feasibility, and clinical applicability. Nanotechnology mainly regulates the spatial and temporal distribution of rutin and its metabolites, whereas structural modification directly alters physicochemical and metabolic properties. Troxerutin represents one of the few clinically translated derivatives with improved human relevance and reduced dependence on gut microbial activation. However, major translational bottlenecks remain unresolved. Most current studies still focus primarily on parent rutin exposure, while metabolite-resolved pharmacokinetic evidence, human ADME data, long-term safety evaluation, and scalable manufacturing strategies remain insufficient. This limitation is further reflected in the current EFSA assessment status, where no authorized health claims for rutin have been approved due to inadequate human evidence. Future studies should prioritize metabolite-resolved pharmacokinetics, gut microbiota-associated variability, clinically relevant ADME evaluation, and scalable translational strategies. Overall, this review provides a metabolism-guided framework for the rational design and clinical translation of rutin-based functional foods and pharmaceutical formulations.

## Figures and Tables

**Table 1:** Functional classification of Rutin nanodelivery systems based on oral ADME regulation.

Classification	Efficacy Enhancement Parameters	Core ADME Regulatory Mechanism	Applicable Diseases	References
Intact-rutin delivery systems	HT-29 IC_50_: 72.85 → 34.75 μg/mL; Colony formation inhibition: 34% → 93%	Enhances cellular uptake of intact rutin	Human colorectal cancer	[49]
	C_max_ ↑ 1.99-fold; AUC_0–∞_ ↑ 2.77-fold; Enhanced brain targeting	TfR-mediated brain targeting; Prolonged circulation	Brain diseases	[50]
	MDA-MB-231 IC_50_: 43.6 μg/mL (NIR); Apoptosis + necrosis: 58.13%; Photothermal ΔT: 5.73 °C; Hemolysis < 5%	pH-responsive tumor release;	Encapsulation by ion gel method	[51]
	Cancer cell lines IC_50_ ↓ 58–81%; VEGFR-2/EGFR/T790M IC_50_ ↓ 30–68%; Transdermal delivery ↑ 2.39-fold	pH-triggered rapid release at pH 4	Liver cancer	[52]
	Body fat percentage decreased to 17.16%; PPARγ downregulated to70%; UCP1 upregulated by 4 times; Locally targeted adipose tissue	Transdermal delivery bypasses GI first-pass metabolism	High-fat diet-induced obesity	[53]
Retention-enhancing systems	↓ IL-6, TNF-α, ROS, MDA, NO levels; ↑ SOD, GSH-Px activities	Mucoadhesive colonic retention; Passive targeting to inflamed mucosa; Local sustained release	D-galactose-induced skeletal muscle dysfunction	[54]
Metabolism-promoting systems	↑ Hepatic GSH-PX activity; ↓ Serum MSTN, TNF-α, IL-6	Enhances colonic delivery;	Osteoporosis	[55]
Active-metabolite direct delivery systems	T_1/2_ ↑ 1.5-fold (8 h → 12 h); Renal accumulation: 20% ID/g	Direct isoquercitrin delivery; KTP-mediated renal targeting	Type 2 Diabetic Nephropathy	[56]
	Upper GI release < 21%; Colon-targeted delivery > 79%	Direct quercetin delivery	Colorectal Cancer	[57]
	Complete wound healing: 18 d (vs. >18 d free quercetin, >21 d control)	Skin-targeted topical delivery	Acute skin trauma	[58]

Note: ↑, increase; ↓, decrease; →, change from baseline to post-treatment value. ADME, absorption, distribution, metabolism, and excretion; AUC_0–∞_, area under the plasma concentration-time curve from time zero to infinity; C_max_, maximum plasma concentration; HT-29, the human colon adenocarcinoma cell line; GI, gastrointestinal tract; IC50, half maximal inhibitory concentration; ID/g, injected dose per gram of tissue; KTP, kidney-targeting peptide; NIR, near-infrared irradiation; t1/2, elimination half-life; TfR, transferrin receptor; MDA-MB-231, human breast adenocarcinoma cell line; VEGFR-2, vascular endothelial growth factor receptor 2; EGFR, epidermal growth factor receptor; T790M, EGFR T790M mutation; PPARγ, peroxisome proliferator-activated receptor gamma; UCP1, uncoupling protein 1; IL-6, interleukin-6; TNF-α, tumor necrosis factor-α; ROS, reactive oxygen species; MDA, malondialdehyde; SOD, superoxide dismutase; GSH-Px, glutathione peroxidase; MSTN, myostatin.

**Table 2:** Summary of representative studies on rutin nano-delivery.

Type	Target	Model	Human Relevance	Pharmacokinetic Changes	Clinical Translation	References
Nanoliposomes	Colon cancer	HT-29 human carcinoma cells	Very low	Unknown	In vitro study	[49]
Metallic Nanoparticles	Antithrombotic	Human umbilical vein endothelial cell model; Mouse tail thrombosis model;	Very low	Unknown	In vitro study	[59]
Polymeric Nanogels	Anticancer	Human malignant tumor cell lines	Very low	Unknown	In vitro study	[52]
Metal Oxide Nanocarriers	Anti-pathogenic bacterial infection	Bacterial strain in vitro model	Very low	Unknown	In vitro study	[60]
Noble Metal Nanoclusters and Polymeric Targeted Carriers	Anti-breast cancer	Breast cancer cell model	Very low	Unknown	In vitro study	[51]
Targeted Nanoliposomes	Brain diseases	C8-D1A mouse astrocyte cell model; SD rat pharmacokinetic model	Low	C_max_ ↑ 1.99-fold; AUC_0–∞_ ↑ 2.77-fold; t_1/2_ ↑ 1.19-fold; MRT_0–∞_ ↑ 1.26-fold; AUC_0-t_ ↑ 2.58-fold	Animal study	[50]
Rutin Nanoemulsion	Parkinson’s disease	Parkinson’s disease oxidative stress rat model	Very low	C_max_ ↑ 1.9-fold; AUC_0–24 h_ ↑ 1.8-fold; T_max_: 4 h → 2 h	Animal study	[61]
Rutin Nanosuspension	Osteoporosis	Human osteoblast-like MG-63 cell model; Ovariectomized (OVX) female Wistar rat osteoporosis model	Low	AUC_0–∞_ ↑ 1.8-fold; C_max_ ↑ 1.9-fold; T_max_: 4 h → 2 h	Animal study	[62]

Notes: ↑, increase; ↓, decrease; →, change from baseline to post-treatment value. HT-29, the human colon adenocarcinoma cell line. C8-D1A, mouse astrocyte cell line; MG-63, human osteoblast-like cell line; SD, Sprague-Dawley rat; Cmax, maximum plasma concentration; Tmax, time to reach maximum concentration; t_1/2_, elimination half-life; MRT_0–∞_, mean residence time from time zero to infinity; AUC_0–∞_, area under the concentration-time curve from time zero to infinity; AUC_0–t_, area under the concentration-time curve from time zero to the last measurable time point; AUC_0–24 h_, area under the concentration-time curve over 0–24 h. A structured seven-dimension scoring matrix was used to assign human relevance grades, with each dimension scored from 0 to 2 (maximum total score: 14). Grade thresholds: Very low (0–4), Low (5–8), Moderate (9–11), High (12–14). The standardized scoring criteria are provided in Table S1 (Supplementary Materials). Itemized scoring records of rutin nano-delivery systems are summarized in Table S2 (Supplementary Materials). Definition of clinical translation categories: marketed compound, commercially approved medicine available on the market; clinical trial, undergoing human clinical investigation; animal study, pharmacokinetic or pharmacodynamic evaluation conducted on laboratory animals; in vitro study, only cell-level or physicochemical experiments without animal tests; theoretical application, preliminary design without any biological verification.

**Table 3:** Summary of representative studies on structural modification of rutin.

Modification	Representative Derivative	Physicochemical & PK Improvement	Human Relevance	Model	Clinical Translation	References
Glycosylation	Rutin 4”-*O*-α-D-glucopyranoside	Water solubility ↑ 128-fold; PK not characterized	Very low	In Vitro Kinetic Model of CGTase Enzymatic Glycosylation	In vitro study	[64]
Metal Ion Complexation	Rutin-Zn complex	Water solubility ↑ 25-fold; Stable aqueous solution ≥ 7 d; PK not characterized	Very low	Murine thrombosis pharmacodynamic model	In vitro study	[65]
Enzymatic Esterification	Rutin esters	Liposolubility ↑ 36.5-fold; PK not characterized	Very low	Regioselective Esterification Model of Lipase	In vitro study	[66]
Metal Ion Coordination	Oxidovanadium (IV)-rutin complex	DMSO solubility ↑ 250-fold; Stable solution ≥ 14 d; PK not characterized	Very low	HeLa human cervical carcinoma cell model	In vitro study	[67]
Hydroxyethylation	Troxerutin	Water solubility ↑ 15–20-fold; Relative bioavailability ≈ 205.55%; C_max_ ↑ 1.75-fold; AUC ↑ 2.06-fold; Tmax slightly prolonged	High	MDCK cell monolayer transepithelial transport model; In vivo rat pharmacokinetic model	Marketed compound	[68]
Benzylation	Benzylated derivative of rutin	Liposolubility ↑ 42.3-fold; Skin permeability ↑ 6.1-fold; PK not characterized	Very low	Human embryonic lung fibroblast MRC-5 cells	In vitro study	[69]
Cocrystal Formation	Rutin-L-proline cocrystal	Solubility ↑ 20-fold; AUC ↑ 15.6-fold vs. raw rutin; C_max_ ↑ 3.8-fold; AUC_0–10 h_ ↑ 5.6-fold;	Very low	Soproterenol-induced cardiac hypertrophy SD rat model	Animal study	[70]

Notes: ↑, indicates increased level; ≥, represents greater than or equal to; ≈, represents approximately; PK, pharmacokinetics. DMSO, dimethyl sulfoxide; MDCK, Madin-Darby canine kidney cell line. C_max_, peak concentration; AUC, area under the concentration-time curve. A structured seven-dimension scoring matrix was used to assign human relevance grades, with each dimension scored from 0 to 2 (maximum total score: 14). Grade thresholds: Very low (0–4), Low (5–8), Moderate (9–11), High (12–14). The standardized scoring criteria are provided in Table S1 (Supplementary Materials). Itemized scoring records of rutin nano-delivery systems are summarized in Table S3 (Supplementary Materials).Definition of clinical translation categories: marketed compound, commercially approved medicine available on the market; clinical trial, undergoing human clinical investigation; animal study, pharmacokinetic or pharmacodynamic evaluation conducted on laboratory animals; in vitro study, only cell-level or physicochemical experiments without animal tests; theoretical application, preliminary design without any biological verification.

**Table 4:** Comprehensive comparison between nanotechnology and structural modification.

Comparison Dimension	Nanotechnology	Structural Modification
Core Strategy	External carrier encapsulation	Internal molecular modification
ADME regulatory mechanism	Can protect the glycosidic bond from premature hydrolysis, actively promote microbial conversion, or completely bypass microbial and hepatic metabolism depending on carrier design	Modifies solubility, lipophilicity, or molecular recognition; most derivatives retain dependence on gut microbiota hydrolysis (troxerutin is the only exception that partially uncouples absorption from microbial deglycosylation)
Effect on metabolic dependence	Can eliminate (active-metabolite direct-delivery), reduce (intact-rutin delivery), or actively utilize (metabolism-promoting) microbial dependence	Rutin generally does not abolish microbial dependence; troxerutin partially attenuates it
Representative bioavailability improvement	Transferrin-modified liposomes: AUC ↑ 2.77-fold (rat); nanoemulsions: AUC ↑ 1.8-fold; nanosuspensions: AUC ↑ 1.8-fold	Troxerutin: AUC ↑ 2.6-fold; C_max_ ↑ 1.75-fold
Targeting capability	Active targeting, passive targeting and physical targeting are achievable	No inherent tissue-targeting ability; requires conjugation with targeting ligands or combination with nanocarriers for site-specific delivery
Regulatory Status	No rutin nanoformulations approved	Troxerutin approved, others still in preclinical research stage
Application	Central nervous system diseases; solid tumors; chronic diseases requiring sustained release	Oral supplements; topical preparations; chronic venous insufficiency

Notes: ADME, absorption, distribution, metabolism, and excretion. AUC, area under the concentration–time curve; C_max_, peak plasma concentration.

## Data Availability

No new data were created or analyzed in this study. Data sharing is not applicable to this article.

## Supplementary Materials

The following supporting information can be downloaded at: https://www.mdpi.com/article/10.3390/ijms27156543/s1.

## Abbreviations

ACC	Acetyl-CoA carboxylase
ADME	Absorption, Distribution, Metabolism, Excretion
AMPK	AMP-activated protein kinase
AUC	Area under the concentration-time curve
BBB	Blood–brain barrier
BCRP	Breast cancer resistance protein
CLint	Intrinsic clearance
COMT	Catechol-O-methyltransferase
CPT-1A	Carnitine palmitoyltransferase 1A
CVI	Chronic venous insufficiency
DOPAC	3,4-Dihydroxyphenylacetic acid
DMSO	Dimethyl sulfoxide
DSPE-PEG	Distearoylphosphatidylethanolamine-poly (ethylene glycol)
EFSA	European Food Safety Authority
EGFR	Epidermal growth factor receptor
EPR	Enhanced permeability and retention effect
FBPase	Fructose-1,6-bisphosphatase
GSH-Px	Glutathione peroxidase
HEK293	Human embryonic kidney 293 cells
IBD	Inflammatory bowel disease
IC_50_	Half maximal inhibitory concentration
IL-6	Interleukin-6
MAPK	Mitogen-activated protein kinase
MDA	Malondialdehyde
MRT	Mean residence time
MRP2/4	Multidrug resistance-associated protein 2/4
NDA	Panel on Dietetic Products, Nutrition and Allergies
NIR	Near-infrared
NO	Nitric oxide
Pd	Palladium
PPARγ	Peroxisome proliferator-activated receptor gamma
PVA	Polyvinyl alcohol
PVP	Polyvinylpyrrolidone
ROS	Reactive oxygen species
SGLT1	Sodium-glucose linked transporter 1
SOD	Superoxide dismutase
STAT3	Signal transducer and activator of transcription 3
TNF-α	Tumor necrosis factor-α
UCP1	Uncoupling protein 1
UGT	UDP-glucuronosyltransferase
VEGFR	Vascular endothelial growth factor receptor
