# Supplementary material for: A Metabolism-Guided Framework for Selecting Structural Modification or Nano-Delivery Strategies for Oral Rutin
Source: Int J Mol Sci. 2026 Jul 23;27(15):6543. doi: 10.3390/ijms27156543 (PMC13465760; doi:10.3390/ijms27156543)
Supplement: Supplementary file 1 [file ijms-27-06543-s001.zip › ijms-4407306-supplementary.pdf]

Part 1. Standard scoring criteria for seven evaluation dimensions.

Evaluation Dimension	0 points	1 point	2 point
Human clinical/PK date	No human-related data reported	In vitro human cell/ex vivo indirect evidence available	Direct human pharmacokinetic or clinical trial data
Metabolite-resolved PK analysis	No metabolite profiling performed	Partial metabolite identification without quantitative PK	Full quantitative metabolite-resolved pharmacokinetics
Oral *in vivo* PK	No oral *in vivo* pharmacokinetic experiment	Oral PK data only obtained from rodents	Oral PK data across multiple species combined with tissue distribution
Tissue distribution characterization	No tissue distribution investigation	Only plasma concentration monitored without organ data	Multi-organ tissue distribution profiles measured
Safety evaluation	No toxicity or safety assessment	Only acute toxicity/preliminary biocompatibility test	Long-term repeated-dose safety and systemic toxicity evaluation
Scalability/manufacturability	Preparation method only theoretically described; scale-up barriers unresolved	Stable preparation achievable at laboratory scale	Pilot or industrial-scale production validated
Regulatory/clinical status	Pure basic research without translational plan	Preclinical animal research stage	Under clinical trials or commercially marketed

Part 2. Detailed scoring results for rutin nano-delivery systems (corresponding to Table 2 in main text).

Formulation	Human data	Metabolite PK	Oral in vivo PK	Tissue distribution	Safety	Scalability	Reg. status	Total Score	Final Human Relevance Grade
Nanoliposomes	0	0	1	0	1	1	1	4	Very low
Metallic nanoparticles	0	0	0	0	0	1	1	2	Very low
Polymeric nanogels	0	0	1	0	1	1	1	4	Very low
Metal oxide nanocarriers	0	0	0	0	0	1	1	2	Very low
Noble-metal nanoclusters and polymeric targeted carriers	0	0	0	0	0	0	1	1	Very low
Targeted nanoliposomes	0	0	1	2	1	0	1	5	Low
Rutin nanoemulsion	0	0	1	0	1	1	1	4	Very low
Rutin nanosuspension (RUT-NS)	0	0	1	0	1	2	1	5	Low

Note:

Scoring remark for Nanoliposomes: Although the original table marked "macokinetic Changes: Unknown", relevant oral absorption investigations were reported in original references, hence Oral *in vivo* PK = 1 point. Unknown PK changes mainly refer to unclear circulating metabolites rather than complete absence of *in vivo* oral tests.

Part 3. Detailed scoring results for structural modification strategies (corresponding to Table 3 in main text).

Formulation	Human data	Metabolite PK	Oral *in vivo* PK	Tissue distribution	Safety	Scalability	Reg. status	Total Score	Final Human Relevance Grade
Glycosylation (Rutin-4''-O-α-D-glucopyranoside)	0	0	0	0	0	0	1	1	Very low
Metal ion complexation (Rutin-Zn complex)	0	0	0	0	0	1	1	3	Very low
Enzymatic esterification (Rutin esters)	0	0	0	0	0	0	1	1	Very low
Metal ion coordination (Oxidovanadium (IV)-rutin complex)	0	0	0	0	0	0	1	1	Very low
Hydroxyethylation (Troxerutin)	2	1	2	1	2	2	2	12	High
Benzylation (Benzylated derivative of rutin)	0	0	0	0	1	0	1	2	Very low
Cocrystal formation (Rutin-L-proline cocrystal)	0	0	1	0	0	1	1	3	Very low
